# Supplementary material for: Wound Healing, Anti-Inflammatory and Anti-Oxidant Activities, and Chemical Composition of Korean Propolis from Different Sources
Source: Int J Mol Sci. 2024 Oct 22;25(21):11352. doi: 10.3390/ijms252111352 (PMC11547006; doi:10.3390/ijms252111352)
Supplement: Supplementary file 1 [file ijms-25-11352-s001.zip › ijms-3249612-supplementary.pdf]

Supplementary Materials for

Anti-Inflammatory and anti-oxidant activities and chemical composition of Korean propolis from different sources

Submitted by Aman Dekebo[1, 4], Chalshisa Geba[2], Daniel Bisrat[2], Jin Boo Jeong[3], and Chuleui Jung*[4,5]

[1]Department of Applied Chemistry, Adama Science and Technology University, Ethiopia; amandekeb@gmail.com (A.D.)

[2]Deparmtment of Pharmacognosy and Pharmaceutical Chemistry, Addis Ababa University; chaligbegna4@gmail.com (C.G.); danielbisrat@gmail.com (D.B.)

[3]Department of Forest Science, Andong National University, Andong 36729, Republic of Korea; jjb0403@anu.ac.kr (C.J.)

[4]Agricultural Science and Technology Research Institute Andong National University, Republic of Korea; cjung@andong.ac.kr (C.J.)

[5]Department of Plant Medicals, Andong National University, Andong GB 36729, Republic of Korea; cjung@andong.ac.kr (C.J.)

* Correspondence: cjung@andong.ac.kr (C.J.); Tel: 054-820-6191; Fax: 054-820-6320

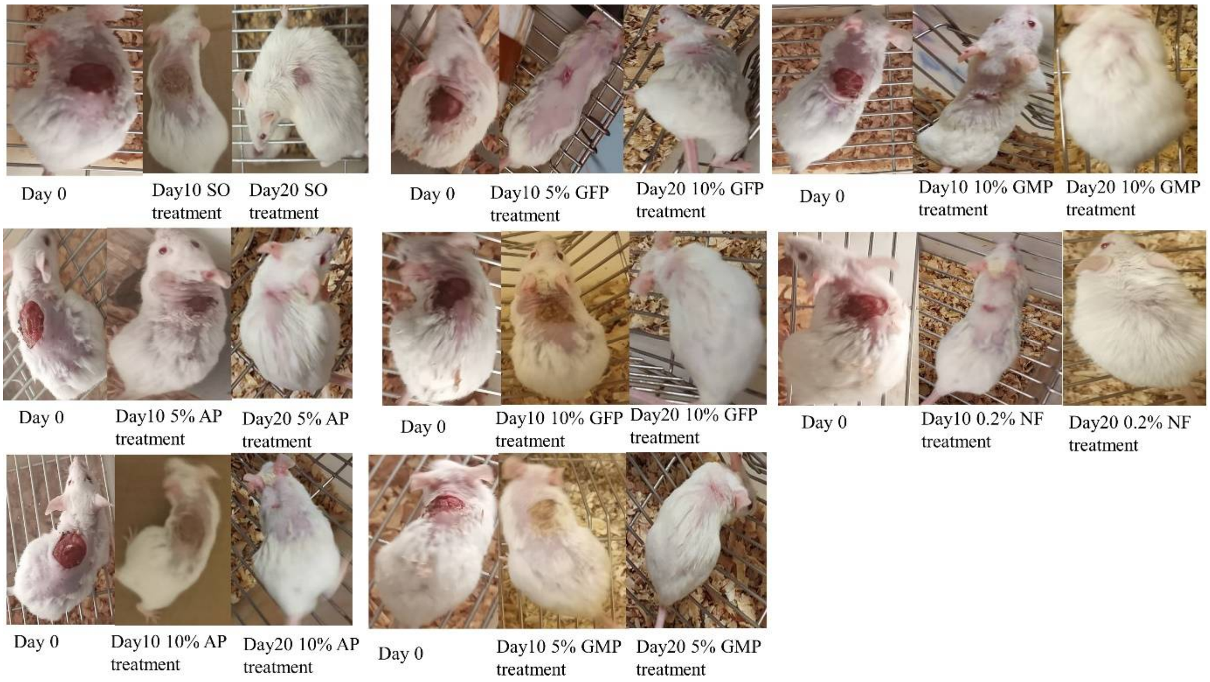

Figure S1. Photograph illustrating the progression of the healing process of excision wounds in mice treated with ethanolic propolis extracts

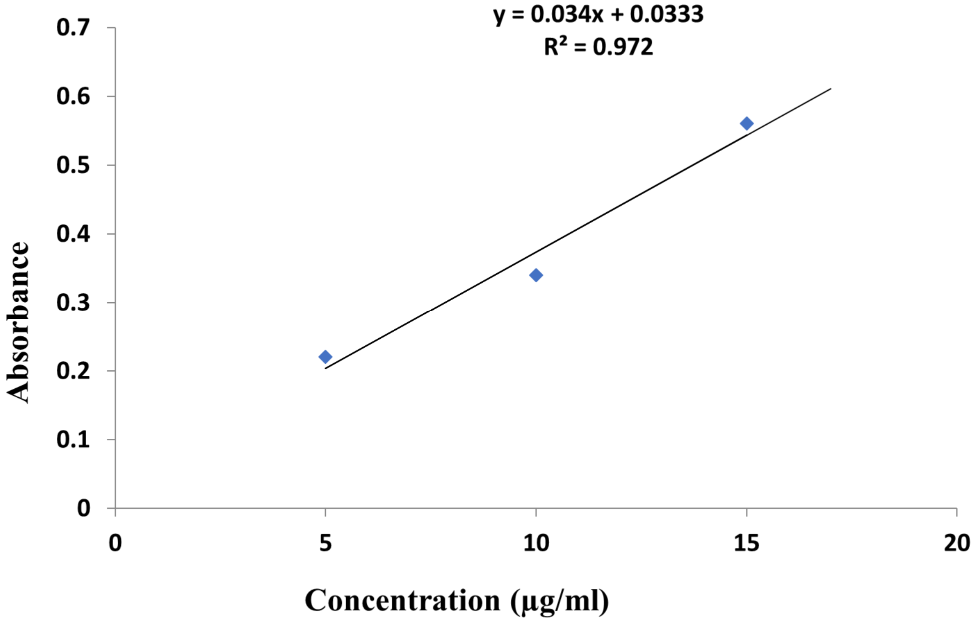

Figure S2. Calibration curve of standard hydroxyproline
